# Analgesia in Minimally Invasive Thoracic Surgery: A Comparison Between Robotic Surgery and Video-Assisted Thoracoscopic Surgery

**DOI:** 10.3390/medicina62071378

**Published:** 2026-07-17

**Authors:** Lucía Valencia, Sara Castillo-Acosta, Ángel Becerra-Bolaños, Carolina Medina, Nazario Ojeda, Aurelio Rodríguez-Pérez

**Affiliations:** 1 Department of Anaesthesiology and Intensive Care, Hospital Universitario de Gran Canaria Doctor Negrín, 35010 Las Palmas de Gran Canaria, Spain; ang_bcr.bol@hotmail.com (Á.B.-B.); cmedram@hotmial.com (C.M.); nojebet@gobiernodecanarias.org (N.O.); arodperp@gobiernodecanarias.org (A.R.-P.); 2 Department of Medicine, University of Fernando Pessoa Canarias, 35010 Las Palmas de Gran Canaria, Spain; 3 Instituto de Investigación Sanitaria de Canarias (IISC), 35010 Las Palmas de Gran Canaria, Spain; 4 Department of Thoracic Surgery, Hospital Universitario de Gran Canaria Doctor Negrín, 35010 Las Palmas de Gran Canaria, Spain; scastilloaco@gmail.com; 5 Department of Medical and Surgical Sciences, Universidad de Las Palmas de Gran Canaria, 35010 Las Palmas de Gran Canaria, Spain

**Keywords:** postoperative pain, robotic-assisted thoracoscopic surgery, minimally invasive surgery

## Abstract

*Background and Objectives*: The recent adoption of RATS (robot-assisted thoracic surgery) alongside VATS (video-assisted thoracoscopic surgery) in minimally invasive thoracic surgery highlights the need for comparative evaluation of both techniques regarding postoperative pain and clinical outcomes. This study compared acute postoperative pain within the first 24 h, as well as postoperative complications, 30-day mortality, and length of hospital and ICU stay. *Materials and Methods*: A retrospective observational study was conducted including all patients scheduled for VATS or RATS at a tertiary hospital between November 2021 and December 2024. Demographic characteristics, surgical procedures, surgical approach, and pain-related outcomes at 24 h (Numeric Rating Scale [NRS], subjective assessment scale, and rescue analgesia) were obtained from the Acute Pain Unit database of the Department of Anesthesiology. Other variables were collected from the electronic medical record. *Results*: A total of 148 patients were analyzed, of whom 118 underwent VATS and 30 RATS. Surgical duration was significantly longer in the RATS group (130 vs. 218 min, *p* < 0.05). No significant differences were observed in NRS scores (2.57 ± 1.06 vs. 2.3 ± 0.79, *p* = 0.195) or subjective pain assessment (good: 78% vs. 83.3%, *p* = 0.472). RATS required less rescue analgesia in the unadjusted analysis (30.0% vs. 52.5% in VATS, *p* = 0.022); however, this association was no longer statistically significant after multivariable adjustment (VATS: OR 2.40, 95% CI 0.93–6.25; *p* = 0.071). There were no significant differences in postoperative complications (17.8% in VATS vs. 16.7% in RATS, *p* = 0.85), length of hospital stay (4.9 ± 6.2 days in VATS vs. 3.4 ± 3 days in RATS, *p* = 0.2), or 30-day mortality (0.8% in VATS vs. 0% in RATS, *p* = 1). ICU length of stay was longer in the RATS group (0.32 ± 0.78 days in VATS vs. 0.73 ± 1.23 days in RATS, *p* = 0.024). *Conclusions*: RATS did not demonstrate superiority over VATS in terms of postoperative pain, patient satisfaction, or clinical outcomes.

## 1. Introduction

Lung cancer is the leading cause of cancer-related mortality in Europe [1]. Pulmonary resection surgery remains the treatment of choice with curative intent in most cases. However, thoracotomy as a surgical approach is associated with a high risk of postoperative complications. One of the main challenges following thoracotomy is the management of acute postoperative pain, which is often severe. Inadequate pain control contributes to pulmonary complications such as atelectasis and pneumonia and is associated with prolonged hospital stays. Moreover, thoracotomy has been linked to a high incidence of chronic post-thoracotomy pain, with some studies reporting an incidence of up to 44% up to one year postoperatively [2].

Minimally invasive thoracic surgery has progressively supplanted open thoracotomy as the preferred approach for pulmonary resections. In fact, in the United States, according to a 2023 review of the Society of Thoracic Surgeons General Thoracic Surgery Database (STS-GTSD), more than 80% of lobectomies, segmentectomies, and wedge resections were performed using minimally invasive techniques, including video-assisted thoracoscopic surgery (VATS) and robotic-assisted thoracic surgery (RATS) [3]. In Europe, the adoption of minimally invasive thoracic surgery has also expanded, although at a slightly slower pace than in the United States. According to a recent survey conducted among members of the European Society of Thoracic Surgeons (ESTS), 68.8% of thoracic surgical procedures were performed using minimally invasive techniques, with the remaining cases still relying on open thoracotomy [4].

This trend toward minimally invasive thoracic surgery over traditional thoracotomy is largely driven by the numerous advantages associated with less invasive approaches. For several years, it has been well established that VATS is associated with significantly less postoperative pain compared to conventional thoracotomy [5,6]. More recently, RATS has emerged as an alternative minimally invasive approach, aiming to further enhance the outcomes achieved with VATS. One of the theoretical advantages of RATS lies in its use of articulated robotic arms that operate around a fixed pivot point. This mechanical configuration may reduce torque on the thoracic wall, thereby minimizing intercostal nerve injury and trauma to surrounding tissues, which could result in decreased postoperative pain. Nevertheless, current evidence remains inconclusive regarding whether RATS offers superior analgesic outcomes compared to VATS.

The aim of this study was to compare acute postoperative pain in patients undergoing minimally invasive thoracic surgery using RATS versus VATS. Additionally, we evaluated differences in clinical outcomes, including morbidity, mortality, and length of hospital stay.

## 2. Materials and Methods

Following approval by the Ethics Committee of Hospital Universitario de Gran Canaria Dr. Negrín on 25 March 2025 (approval No. CEI: 2025-109-1), we conducted a retrospective cohort study. Inclusion criteria were patients aged 18 years and older, scheduled for oncologic thoracic minimally invasive surgery (RATS and VATS) between November 2021 and December 2024. Exclusion criteria were conversion to thoracotomy.

### 2.1. Routine Peri-Procedural Management

All patients underwent thoracic oncological surgery under general anaesthesia. Surgical procedures included lobectomies, segmentectomies, and wedge resections. Wedge resections were mainly performed for diagnostic purposes in patients with suspicious pulmonary lesions or for the resection of metastatic disease. VATS procedures were performed using a uniportal or biportal approach, with an incision smaller than 5 cm, typically accompanied by an accessory trocar, and without rib spreading. RATS procedures were performed using a four-arm technique with an additional 12-mm port for the bedside assistant. All RATS procedures were performed by a single console surgeon, who had five years of experience as a thoracic surgeon, assisted by a bedside assistant. The other two thoracic surgeons involved in the study performed VATS procedures throughout the study period, maintaining the same surgical team composition, whereas RATS procedures were consistently performed by the same console surgeon.

Patients were premedicated with intravenous midazolam (1–2 mg) and monitored using non-invasive blood pressure, electrocardiography, pulse oximetry (SpO_2_), and bispectral index monitoring (BIS; Covidien, Dublin, Ireland). General anaesthesia was maintained with either sevoflurane or propofol at the discretion of the attending anaesthesiologist. Cisatracurium was administered as an initial bolus (0.2 mg/kg) to facilitate orotracheal intubation, followed by continuous intravenous infusion throughout surgery. Intraoperative fentanyl administration was left to the discretion of the attending anaesthesiologist.

All patients received an intravenous loading dose of the analgesic drugs that were subsequently continued as a continuous infusion during the first 24 postoperative hours. One-lung ventilation was achieved using a VivaSight double-lumen endotracheal tube (VivaSight-DLT, Ambu^®^, Ballerup, Denmark), allowing continuous visualization and verification of correct tube placement. A lung-protective ventilation strategy was applied, with tidal volumes of 6–8 mL/kg predicted body weight and a positive end-expiratory pressure (PEEP) of 5 cm H_2_O.

At the end of surgery, a 16-Fr (5.33 mm) chest tube was placed. Neuromuscular blockade reversal was administered before extubation. No regional anaesthesia techniques were employed.

Patients were transferred to either the Post-Anaesthesia Care Unit (PACU) or the postoperative Intensive Care Unit (ICU), where they were managed by an independent clinician. The decision regarding the postoperative destination was made jointly by the thoracic surgery and anaesthesiology teams, based on the patient’s medical history and the intraoperative course. Patients admitted to the PACU were usually discharged after 6 h, following a chest radiograph confirming the absence of postoperative complications.

In the ICU, the standard length of stay was 24 h. During this period, most patients resumed oral intake and were mobilized to a sitting position on the day of surgery. Transfer to the surgical ward was performed after evaluation of laboratory tests and chest radiography confirming the absence of complications. Chest tubes were removed once any air leak had resolved and pleural drainage was less than 40 mL over a 24-h period.

Postoperative analgesia was administered according to standardized protocols established by the Acute Pain Unit of our hospital, combining different drugs delivered via continuous infusion pumps during the first 48 postoperative hours (Table 1). The choice of analgesic protocol depended on factors such as medical history, allergies, and the patient’s clinical condition. The most used regimen consisted of tramadol combined with metamizole or dexketoprofen (Protocol A). In elderly patients or those with renal impairment, either a reduced dose of tramadol combined with metamizole (Protocol B) or dexketoprofen or metamizole (Protocol C) was used. Continuous morphine infusion was reserved for selected cases, such as patients with chronic opioid use (Protocol D).

### 2.2. Routine Pain Evaluation

The Acute Pain Unit of the Department of Anaesthesiology conducted standardized daily follow-up of postoperative analgesic management. Twenty-four hours after surgery, an anaesthesiologist from the Acute Pain Unit visited each patient on the ward to assess postoperative pain control. Pain intensity was evaluated using the Numeric Rating Scale (NRS), in which patients rated their pain on an 11-point scale ranging from 0 (no pain) to 10 (worst imaginable pain). Based on the NRS score, postoperative pain was categorized as mild (NRS 1–3), moderate (NRS 4–7), or severe (NRS 8–10).

Patient satisfaction with postoperative analgesia was assessed using a Subjective Assessment Scale (SAS). Patients were asked to rate their satisfaction with pain control as excellent, good, fair, or poor. In addition, the number of rescue analgesic interventions was recorded in cases in which patients reported an NRS score >3 despite receiving protocolized analgesia. In these cases, first-rescue analgesia was administered, and if pain remained >3 on the NRS 20 min later, second-rescue analgesia was provided according to the institutional protocol. Following the daily assessment, the attending anaesthesiologist from the Acute Pain Unit recorded all data in the unit’s database.

### 2.3. Data Collection

Patients included in the study were identified through the Acute Pain Unit, part of the Department of Anaesthesia, and the Postoperative Intensive Care Unit database. Pain-related data were collected from this database and included analgesic protocols, SAS and NRS scores, as well as the percentage and number of rescue analgesic interventions.

Additional variables were obtained from the electronic medical records and included demographic and clinical data such as body mass index (BMI), ASA physical status, and medical history, including smoking status, hypertension, ischaemic heart disease, depression, chronic obstructive pulmonary disease (COPD), and preoperative forced expiratory volume in 1 s (FEV1). Surgical variables included the surgical approach (RATS or VATS), type of surgery (lobectomy, segmentectomy, or wedge resection), and intraoperative duration. Postoperative variables included complications defined according to the Clavien–Dindo classification, length of stay in the PACU/ICU and overall hospital stay, hospital readmission within 30 days, and mortality at 30 days and 1 year.

### 2.4. Statistical Analysis

Categorical variables were expressed as frequencies and percentages. Comparisons between groups were performed using the chi-square test or Fisher’s exact test, as appropriate. Continuous variables were expressed as mean ± standard deviation (SD). Normality of data distribution was assessed using the Shapiro–Wilk test.

For comparisons of continuous variables between two groups, the independent samples *t*-test was used for normally distributed variables, whereas the Mann–Whitney U test was applied when normality assumptions were not met. For comparisons among more than two groups, one-way analysis of variance (ANOVA) was used for normally distributed variables, and the Kruskal–Wallis test was applied for non-normally distributed data. Univariate logistic regression analyses were performed to identify factors related to the need for rescue analgesia. Factors with *p*-values < 0.05 in the univariate logistic regression model, as well as those considered clinically important, were evaluated by multiple logistic regression analysis. Odds ratios and 95% confidence intervals were estimated, and *p*-values < 0.05 were considered statistically significant.

A *p* value < 0.05 was considered statistically significant. No formal sample size calculation was performed because of the retrospective design; therefore, the study should be considered exploratory. Statistical analyses were performed using SPSS Statistics version 24.0 (IBM Corp., Armonk, NY, USA).

## 3. Results

During the study period, 164 patients underwent minimally invasive surgery. Sixteen patients were excluded due to conversion to thoracotomy secondary to technical difficulties. Finally, 148 patients were included in the analysis: 118 underwent VATS and 30 underwent RATS. The baseline characteristics of the overall population, as well as those of both groups, are presented in Table 2. Intraoperative variables are also summarized in Table 2.

The analgesic regimen remained unchanged during the observation period, and no adjustments were required based on pain control. No significant differences were observed in analgesic protocols according to the surgical approach (Table 3). Up to 66% of patients received a combination of tramadol and dexketoprofen/metamizole. Morphine was reserved for breakthrough pain in both the VATS and RATS groups.

Regarding postoperative pain assessment at 24 h, results were similar between the two surgical approaches. However, the need for rescue analgesia, both in terms of the percentage of patients and the total number of interventions, was higher in the VATS group compared to the RATS group, and these differences were statistically significant (Table 4).

To identify factors associated with the need for rescue analgesia, univariate logistic regression analyses were initially performed. Variables with a *p*-value < 0.05 in the univariate analysis, together with clinically relevant variables, were subsequently included in a multivariable logistic regression model (Table 5). Surgical approach, sex, and length of surgery were entered into the final model. In the multivariable model including sex, surgical approach and operative time, surgical approach was associated with a trend toward lower rescue analgesia requirements, although statistical significance was not reached (OR 2.4, *p* = 0.071).

When comparing postoperative data, outcomes were similar between groups, except for PACU/ICU stay, which was longer after RATS than after VATS (Table 6).

## 4. Discussion

In our study, no significant differences were observed in acute postoperative pain between VATS and RATS. In the unadjusted analysis, patients undergoing RATS required less rescue analgesia during the first 24 h; however, this association was not confirmed after multivariable adjustment.

Nowadays, there is increasing emphasis on the need to improve patient recovery after surgery by reducing surgical stress, complications, and length of hospital stay, without increasing risk through Enhanced Recovery After Surgery (ERAS). Consequently, surgical techniques have evolved toward minimally invasive approaches. In thoracic surgery, this has led to the development of VATS and, more recently, RATS.

Thoracic surgeons clearly recognize the technical advantages of robotic-assisted surgery, including superior visualization, enhanced precision, and improved instrument maneuverability, and there is strong interest among them in acquiring the necessary training to adopt this approach. However, its limited dissemination is largely related to concerns about cost-effectiveness and the need for dedicated training, particularly given the limited availability of RATS simulators. In this context, it becomes essential to critically assess whether these theoretical advantages translate into meaningful clinical benefits and to determine whether the adoption of this more expensive technology—requiring additional surgeon training—is truly justified.

One of the most critical aspects in thoracic surgery is the management of postoperative pain, which can often be challenging. Even after VATS procedures, patients may experience significant pain in the early postoperative period. The incidence of moderate-severe pain was 12.7% within the first 24 h and 15.6% within the first 48 h after surgery [7]. Particularly notable is the high incidence of chronic post-thoracotomy pain, which varies among studies from 16.2% [8] to 43% [9].

RATS is theoretically expected to result in less acute postoperative pain, as intercostal nerve injury appears to be reduced by aligning the remote center of the trocars with the midpoint of the chest wall [10]. Several studies have been conducted with the aim of comparing the incidence of chronic pain at six months between both surgical approaches. They do not demonstrate significant differences between VATS and RATS [11,12].

However, one of the most important risk factors for the development of chronic postoperative pain is the presence of acute postoperative pain following thoracic surgery [13]. Our study focuses on acute pain within the first 24 h, comparing the VATS and RATS surgical approaches. In a large prospective multicenter study conducted in Japan [10], postoperative pain was assessed. However, evaluations were not performed during the immediate postoperative period, but rather at 10, 30, and 90 days after surgery. The proportion of patients with an NRS score ≤ 3 was higher in the RATS group.

To date, few studies have specifically evaluated acute postoperative pain within the first 24 h following VATS and RATS, and they have not consistently demonstrated reduced pain levels in patients undergoing RATS [14,15]. In contrast, a more recent retrospective study has reported lower pain levels during the early postoperative period in patients undergoing VATS [16].

There are several reasons why previous studies have failed to demonstrate that RATS is associated with lower postoperative pain. First, the method used to assess pain may play a significant role. Many of these studies rely exclusively on the Numeric Rating Scale (NRS) [14,17] or on postoperative analgesic consumption [18] as outcome measures, which may impair an accurate assessment of acute postoperative pain.

The NRS is a validated, simple, and widely used tool for pain assessment. The Visual Analogue Scale (VAS) is also commonly used but NRS is more practical and easier to understand for most patients than VAS. The NRS and VAS have been shown to yield nearly identical values in the same patient at various times after surgery, and therefore can be used interchangeably [19]. Currently, NRS is recommended by a multisociety organizational consensus for acute perioperative pain management [20].

However, its value is limited when used as the sole measure of pain. For example, when the NRS is used to estimate pain over a broader time frame (e.g., the previous 24 h), it may fail to capture transient pain exacerbations experienced by the patient and therefore may not accurately reflect the temporal variability of postoperative pain. In our study, we evaluated rescue analgesia requirements to achieve a more precise assessment of postoperative pain within the first 24 h. Although no significant differences were observed in NRS scores or patient satisfaction, a lower requirement for rescue analgesia was observed in the RATS group in the unadjusted analysis. However, this association was not maintained after multivariable adjustment, and therefore its clinical significance should be interpreted with caution. Further studies are needed to determine whether RATS may provide meaningful advantages in postoperative pain management. We also used a categorical pain scale, but only in combination with the NRS, since the verbal categories “mild,” “moderate,” and “severe” pain may correspond to different values on the NRS in the same patient. The use of multiple pain assessment scales, with the NRS serving as the primary measurement, may represent the most effective approach for accurately managing postoperative pain.

The literature also recommends incorporating functional variables to assess the impact of pain on an individual’s ability to perform activities of daily living, participate in physical therapy, and evaluate the progression of functional status after surgery [20]. However, these scales may be more appropriate beyond the immediate postoperative period, rather than within the first 24–48 h.

A second factor that may have contributed to the lack of superior postoperative pain outcomes with RATS in previous studies is the longer operating time compared with VATS, which may be associated with worse postoperative outcomes. In the meta-analyses published [21,22], no significant differences have been found between RATS and VATS regarding surgery time. However, these meta-analyses are recent and predominantly include studies from the United States, where experience with the da Vinci robotic system exceeds twenty years. Similar to our findings, earlier studies from the United States [23], where experience was still limited, have reported a longer duration of RATS compared with VATS, attributing this finding to both the time required for robot docking and the surgical team’s learning curve. These factors may also explain our results, as RATS represents a relatively new technique at our center and is still in the implementation phase. With increasing surgical experience and progression beyond the initial learning curve, operative times may potentially approach those of VATS, as has been reported in high-volume centers. However, this potential improvement remains to be confirmed in future studies.

In our study, no statistically significant differences were observed in postoperative morbidity between the two groups. Although a slightly higher number of complications classified as Clavien–Dindo grade III and IV were recorded in the RATS group, this difference did not reach statistical significance. Kwon et al. [14] reported a statistically significant increase in postoperative urinary retention in patients undergoing RATS compared with VATS, a complication that the authors attributed to longer surgery and anaesthetic times.

In contrast to our findings, other studies have reported lower morbidity associated with the robotic approach. Postoperative atrial fibrillation is a common complication after thoracic surgery, with an estimated incidence of approximately 15%. This is mainly due to the proximity of structures such as the pericardium and the pulmonary hilum. Several studies [15,24] have found a statistically significant difference in cardiovascular complications, with a higher incidence of postoperative arrhythmias (mainly atrial fibrillation) in the VATS group.

On the other hand, when comparing ICU length of stay, a longer duration was observed in patients undergoing RATS. This finding may be related to the longer operative duration associated with this approach, which could have led to a more cautious postoperative monitoring strategy. In contrast, overall hospital stay was similar between groups and was even shorter in the RATS group, with fewer readmissions, although without statistically significant differences. In many studies [21], robotic surgery has shown slightly shorter hospital stays compared with VATS. This may be due to greater surgical precision and improved visualization during the procedure, which may facilitate faster recovery in the RATS approach.

No differences in 30-day mortality were observed between RATS and VATS, with both minimally invasive approaches demonstrating similarly low perioperative mortality rates [24]. These findings are consistent with previous literature. Some authors have suggested potential oncological advantages of RATS, including more extensive lymphadenectomy; however, these results should be interpreted with caution given the lack of statistically significant differences in our analysis. In our study, higher 1-year mortality was observed in the VATS group (13%) compared with the RATS group (6%), although the difference was not statistically significant. These mortality rates should be interpreted within the context of an oncological population. Although specific causes of death were not available for analysis, no deaths were directly related to postoperative complications, and long-term mortality is unlikely to be attributable to the surgical approach itself.

The study is limited by its retrospective design and the imbalance in sample size between groups, with a smaller RATS cohort, which may reduce statistical power and limit the generalizability of the findings. In addition, no formal sample size calculation was performed, and the limited number of RATS patients may have reduced the ability to detect clinically relevant differences in some outcomes, including pain, complications, and mortality. A second limitation is the absence of long-term follow-up: given the high incidence of chronic postoperative pain after thoracic surgery, a 3-month telephone follow-up would have been valuable to better assess its prevalence. Additionally, the lack of a standardized analgesic protocol between the two groups represents another important limitation, as variability in postoperative pain management may have influenced pain outcomes and reduced the accuracy of intergroup comparisons. Rescue analgesia should also be interpreted cautiously, as it may be influenced by the initial analgesic protocol, patient-specific factors, and clinical practice patterns. Furthermore, although postoperative pain is generally most intense during the first 24 h after surgery and this time point was therefore selected for analysis, the inclusion of serial pain assessments, particularly at 48 h, would have provided a more comprehensive characterization of postoperative pain trajectories. Finally, the absence of regional analgesia techniques in both groups should be acknowledged. Although regional analgesia is commonly used in thoracic ERAS pathways, our institution achieves adequate postoperative pain control through a standardized multimodal analgesic strategy, including continuous analgesic infusion pumps and close monitoring by the Acute Pain Service. Therefore, the present findings may not be directly generalizable to institutions where paravertebral block, erector spinae plane block, intercostal nerve block, or other regional analgesic techniques are routinely used.

## 5. Conclusions

In conclusion, although RATS has not demonstrated definitive advantages in our population, further experience and completion of the learning curve may potentially contribute to reducing operative times and optimizing perioperative outcomes. However, these potential benefits remain to be confirmed in future studies with larger cohorts and longer follow-up.

## Figures and Tables

**Table 1:** Analgesic Protocols administered intravenously postoperatively.

Analgesic Protocols	Drugs Administered During 48 h
Protocol A	Tramadol 300–400 mg + metamizole 12 g or dexketoprofen 300 mg + antiemetic (metoclopramide 60 mg) First rescue: acetaminophen intravenous 1 g every 8 h Second rescue: Morphine 3 mg subcutaneously every 6 h
Protocol B	Tramadol (150 mg) + metamizole 12 g + antiemetic (metoclopramide 60 mg) First rescue: acetaminophen 1 g intravenous every 8 h Second rescue: Morphine 3 mg subcutaneously every 6 h
Protocol C	Metamizole 12 g or dexketoprofen 300 mg First rescue: acetaminophen intravenous 1 g every 8 h Second rescue: Tramadol 50 mg intravenous every 8 h
Protocol D	Morphine PCA + metamizole 2 g every 8 h First rescue: acetaminophen 1 g intravenous every 8 h

PCA: patient-controlled analgesia.

**Table 2:** Comparison of preoperative characteristics of patients and intraoperative data between RATS or VATS.

		Study Population (*n* = 148)	VATS (*n* = 118)	RATS (*n* = 30)	*p* Value
Age, years		59 ± 14.39	59.07 ± 15.37	62.23 ± 9.41	0.284
Male, *n* (%)		80 (54.1%)	61 (51.7%)	19 (63.3%)	0.253
BMI, kg·m^−1^			24.6 ± 4.7	25.0 ± 6.1	0.767
Smoker, *n* (%)					
	Non smoker	49 (33.1%)	40 (34.2%)	9 (30%)	0.909
	Current-Smoker	38 (25.6%)	30 (25.4%)	8 (26.7%)	
	Ex-smoker	61 (41.2%)	48 (41%)	13 (43.3%)	
ASA physical status, *n* (%)					
	I	4 (2.7%)	4 (3.4%)	0 (0%)	0.599
	II	37 (25%)	29 (24.6%)	8 (26.7%)	
	III	104 (70.2%)	83 (70.3%)	22 (73.3%)	
	IV	3 (2%)	3 (2.5%)	0 (0%)	
Medical history					
	Arterial hypertension, *n* (%):	70 (47.3%)	54 (45.8%)	16 (53.3%)	0.458
	Ischaemic heart disease, *n* (%)	13 (8.8%)	9 (7.6%)	4 (13.3%)	0.324
	COPD, *n* (%)	70 (47.3%)	54 (45.8%)	16 (53.3%)	0.458
	Depression, *n* (%)	11 (7.4%)	7 (5.9%)	4 (13.3%)	0.168
	Preoperative FEV_1_ (% predicted)	62.71 ± 42.01	60.03 ± 43.06	73.27 ± 36.35	0.124
Length of surgery, min		148.16 ± 96.89	130.2 ± 86.29	218 ± 105.37	0.001
Type of surgery, *n* (%)					
	Lobectomy	25 (16.8%)	20 (16,9%)	5 (16,6%)	0.125
	Segmentectomy	63 (42.5%)	50 (42.3%)	13 (43.3%)	
	Wedge resection	60 (40.5%)	48 (40.6%)	12 (40%)	

Data are expressed as mean + SD, or frequency (%). RATS: robotic-assisted thoracic surgery; VATS: video-assisted thoracic surgery; BMI: body mass index; ASA: American Society of Anesthesiologists; COPD: Chronic Obstructive Pulmonary Disease.

**Table 3:** Analgesic Protocols According to Surgical Approach.

Analgesic Protocol, *n* (%)	Study Population (*n* = 148)	VATS (*n* = 118)	RATS (*n* = 30)	*p* Value
Protocol A: Tramadol 300–400 mg + metamizole 12 g or dexketoprofen 300 mg + antiemetic (metoclopramide 60 mg) during 48 h.	88 (59.5%)	70 (59.3%)	18(60%)	0.140
Protocol B: Tramadol 150 mg + metamizole 12 g + antiemetic (metoclopramide 60 mg) during 48 h.	10 (6.7%)	6 (5.1%)	4 (13.3%)	
Protocol C: Metamizole 12 g or dexketoprofen 300 mg during 48 h.	48 (32.4%)	40 (33.8%)	8 (26.7%)	
Protocol D: Morphine PCA + metamizole 2 g	2 (1.35%)	2 (1,7%)	0 (0%)	

Data are expressed as frequencies (%) or mean + SD. RATS: robotic-assisted thoracic surgery; VATS: video-assisted thoracic surgery; PCA: patient-controlled analgesia.

**Table 4:** Twenty-four-hour pain assessment according to surgical approach (VATS vs. RATS).

	Study Population (*n* = 148)	VATS (*n* = 118)	RATS (*n* = 30)	*p* Value
NRS category, *n* (%)				
1–3	126 (85.1%)	98 (83%)	28 (93.3%)	0.170
4–7	22 (14.8%)	20 (16.9%)	2 (6.6%)	
8–10	0	0	0	
NRS score	2.52 ± 1.01	2.57 ± 1.06	2.3 ± 0.79	0.195
SAS, *n* (%)				
Excellent	12 (8.1%)	9 (7.6%)	3 (10%)	0.472
Good	117 (79.1)	92 (78%)	25 (83.3%)	
Fair	16 (10.8)	15 (12.7%)	1 (3.3%)	
Poor	3 (2%)	2 (1.7%)	1 (3.3%)	
Number of additional analgesic requirements	0.87 ± 1.16	1 ± 1.23	0.37 ± 0.67	0.007
Rescue analgesia, *n* (%)	71 (48%)	62 (52.5%)	9 (30%)	0.022

Data are expressed as frequencies (%) or mean + SD; RATS: robotic-assisted thoracic surgery; VATS: video-assisted thoracic surgery; NRS: Numeric Rating Scale; SAS: Subjective Assessment Scale.

**Table 5:** Univariate and multivariate logistic regression analyses of factors predictive of rescue analgesia.

Predictors	Univariate Analysis		Multivariate Analysis	
	OR (95%CI)	*p*-Value	OR (95%CI)	*p*-Value
Female	3.59 (1.82–7.1)	0.001	3.42 (1.71–6.88)	0.001
Age	0.99 (0.96–1.01)	0.461		
ASA	0.7 (0.36–1.28)	0.248		
Type of surgery	1.05 (0.93–1.2)	0.375		
Surgical approach (VATS)	2.51 (1.09–6)	0.031	2.4 (0.927–6.25)	0.071
Length of surgery	0.99 (0.99–1)	0.279	1 (0.996–1)	0.963
Analgesic protocol	0.9 (0.73–1.04)	0.182		

Data are expressed as OR: odds ratio; CI: confidence interval; ASA: American Society of Anesthesiologists; VATS: video-assisted thoracic surgery.

**Table 6:** Length of ICU and hospital stay, and morbidity and mortality rates according to the surgical approach (VATS vs. RATS).

		Study Population (*n* = 148)	VATS (*n* = 118)	RATS (*n* = 30)	*p* Value
PACU/ICU stay, days		0.4 ± 0.9	0.32 ± 0.78	0.73 ± 1.23	0.024
Hospital length, days		4.6 ± 5.6	4.91 ± 6.2	3.43 ± 3.08	0.202
Hospital readmissions within one month, *n* (%)		4 (2.7%)	4 (3.4%)	0 (0%)	0.307
Mortality at 30 days, *n* (%)		1 (0.6%)	1(0.8%)	0	1
Mortality at 1 year, *n* (%)		18 (12.1)	16 (13.6%)	2 (6.7%)	0.302
Postoperative complications, *n* (%)		26 (17.5)	21 (17.8%)	5 (16.7%)	0.885
	Clavien-Dindo grade I and II	13 (8.7%)	11 (9.3%)	2 (6.7%)	0.646
	Clavien-Dindo grade III and IV	13 (8.7%)	10 (8.6%)	3 (10%)	0.813

Data are expressed as frequencies (%) or mean + SD. RATS: robotic-assisted thoracic surgery; VATS: video-assisted thoracic surgery; PACU: Post-Anesthesia Care Unit; ICU: Intensive Care Unit.

## Data Availability

The original contributions presented in this study are included in the article. Further inquiries can be directed to the corresponding author.

## Abbreviations

The following abbreviations are used in this manuscript:

RATS	Robot-assisted thoracic surgery
VATS	video-assisted thoracoscopic surgery
NRS	Numeric Rating Scale
ICU	Intensive Care Unit
PACU	Post-Anesthesia Care Unit
PCA	Patient-controlled analgesia
VAS	Visual Analogue Scale
